# The correlation between antibiotic usage and antibiotic resistance: a 3-year retrospective study

**DOI:** 10.3389/fcimb.2025.1608921

**Published:** 2025-08-01

**Authors:** Ann Lisa Arulappen, Amer Hayat Khan, Syed Shahzad Hasan, Sabariah Noor Harun, Ting Soo Chow, Mahmood Basil A. Al-Rawi, Wajid Syed

**Affiliations:** ^1^ Discipline of Clinical Pharmacy, School of Pharmaceutical Sciences, University Science Malaysia, Georgetown, Malaysia; ^2^ Department of Pharmacy, Penang Hospital, Ministry of Health, George, Town, Malaysia; ^3^ Department of Pharmacy, School of Applied Science, University of Huddersfield, Huddersfield, United Kingdom; ^4^ Department of Medicine, Penang Hospital, Ministry of Health, Georgetown, Malaysia; ^5^ Department of Optometry, College of Applied Medical Sciences, King Saud University, Riyadh, Saudi Arabia; ^6^ Department of Clinical Pharmacy, College of Pharmacy, King Saud University, Riyadh, Saudi Arabia

**Keywords:** antimicrobial resistance, antibiogram, gram positive, gram negative, prevalence, antibiotic usage

## Abstract

**Introduction:**

Multidrug-resistant (MDR) microorganisms have increased all over the world, which is considered a public health threat. The emergence of MDR bacterial pathogens correlates with the increased antibiotic usage. This study aimed to determine the correlation between antibiotic usage and antibiotic resistance within 3 years.

**Method:**

This was a retrospective cross-sectional study reviewing the positive bacterial culture results and the total antibiotic usage in six hospitals in Penang for 3 years from January 2021 to December 2023 through a convenient sampling method.

**Results:**

Every sample type has experienced a significant shift over the years. Most microorganisms from all samples significantly changed in distribution over time, except for *Streptococcus pneumoniae*, carbapenem-resistant Enterobacterales (CRE) *Escherichia coli*, and CRE *Klebsiella pneumoniae*. However, methicillin-resistant *Staphylococcus aureus* (MRSA), *K. pneumoniae*, and *Pseudomonas aeruginosa* showed significant changes in the number of total isolates from blood cultures only in the 3 years. In terms of prevalence, statistically significant differences were observed for most microorganisms from all samples except for *S. pneumoniae*, CRE *E. coli*, and CRE *K. pneumoniae* across the years. *P. aeruginosa* showed significant prevalence in blood culture over time. Cefoperazone/sulbactam, amoxicillin/clavulanic acid, ceftriaxone, and ceftazidime showed significant changes in susceptibility for *K. pneumoniae* over time. A statistically significant difference in total antibiotic usage across the 3 years was observed. Regarding the correlation between antibiotic usage and antibiotic resistance, Pearson’s correlation was 0.777 (p = 0.433), which is suggestive of a strong positive correlation between third-generation cephalosporin usage and extended-spectrum beta-lactamase (ESBL), whereas Pearson’s correlation was 0.762 (p = 0.448), which also suggests strong positive correlation between carbapenem usage and CREs.

**Conclusion:**

The correlation between the use of third-generation cephalosporins and ESBL rate, as well as the use of carbapenems and CRE rate, further suggests that controlling certain antibiotic usage could help mitigate the rise in MDR microorganisms.

## Introduction

Multidrug-resistant (MDR) microorganisms have increased all over the world, which is considered a public health threat. Several recent investigations have reported the emergence of multidrug-resistant bacterial pathogens from different origins, increasing the necessity of the judicious use of antibiotics, in addition to the routine application of antimicrobial susceptibility testing to detect the appropriate antibiotic of choice, as well as the screening of the emerging MDR strains. Antimicrobial resistance occurs when microbes like bacteria and fungi develop mechanisms to withstand the effects of drugs designed to kill them. Infections caused by resistant bacteria can be difficult, and sometimes impossible, to treat. Antimicrobial resistance is a naturally occurring process. However, increases in antimicrobial resistance are driven by a combination of microbes exposed to antibiotics and antifungals and the spread of those microbes and their resistance mechanisms. Alarmingly, antimicrobial-resistant germs can share their resistance mechanisms with other microbes that have not been exposed to antibiotics or antifungals. Antibiotic-resistant strains originating from animals can also be disseminated to humans through the food supply, by direct contact with the animals, or through environmental routes (Lhermie et al., 2016; Qureshi et al., 2024; Shami et al., 2024). In addition, several researchers have suggested an association between antimicrobial usage and the presence of antimicrobial-resistant strains found not only in livestock but also in human-to-human transmission (Sharma et al., 2017). A study conducted in slaughterhouses has revealed that all *Pseudomonas aeruginosa* isolates were MDR, most of them have multiple resistant genes, including the blaCTX-M gene, and some strains have the ability to form biofilms and have virulence genes (Al-Kadmy et al., 2024). Alarmingly, a similar study on birds from various geographical areas found extensive drug-resistant (XDR) *P. aeruginosa* carrying multiple virulence-related genes (Algammal et al., 2023). A study in Egypt reported the isolation of *Staphylococcus aureus* from diseased ducks at a prevalence of >10% (Eid et al., 2019). An *Escherichia coli* strain was isolated in China and found to carry multiple resistance genes that exhibit resistance to almost all clinically used antibiotics (Shafiq et al., 2022).

The World Health Organization (WHO) reported that globally, in 2019, approximately 4.95 million people who died suffered from drug-resistant infections. Antimicrobial resistance (AMR) directly caused 1.27 million of those deaths. The number of deaths attributed to AMR in Malaysia is higher than deaths from digestive diseases, diabetes and kidney diseases, transport injuries, chronic respiratory diseases, and neurological disorders. There are five pathogens to be aware of in Malaysia (number of deaths associated with AMR in parentheses): *Streptococcus pneumoniae* (3,200, 33%), *E. coli* (2,100, 21%), *Acinetobacter baumannii* (1,900, 20%), *Klebsiella pneumoniae* (1,400, 14%), and *S. aureus* (1,200, 12%) (National Antibiotic Resistance Surveillance Report 2023). Antimicrobial resistance in Malaysia continues to escalate and is attributed to the overuse and misuse of antibiotics, including those that can be purchased over the counter. The Malaysian government has come up with an action plan to create public awareness regarding the health implications of antibiotic resistance (Naeemmudeen et al., 2021). Another study from Malaysia had revealed the correlation between 3-year trends of antibiotic consumption [defined daily dose (DDD)/100 admissions] with MDR infection cases (per 100 admissions). The antimicrobial resistance trend demonstrated a positive correlation between extended-spectrum cephalosporins and fluoroquinolones towards the development of resistant microorganisms (Tan et al., 2022).

A key component of antibiotic stewardship programs is the collection and dissemination of antibiotic resistance patterns or antibiograms for various microorganisms. In Malaysia, the antibiogram used is a numerical profile detailing the susceptibility of various microorganisms to multiple antibiotics cultured from patients (William et al., 2021). An antibiotic is deemed susceptible to a specific microorganism if it likely responds to treatment at standard dosing as per the European Committee on Antimicrobial Susceptibility Testing (EUCAST, 2022) recommendation. These data must be regularly updated to keep pace with the rapid emergence of resistant bacterial pathogens (Antibiotic resistance,World Health Organization, 2023). Insights from this study are expected to provide a framework for addressing antibiotic resistance and potentially lowering overall mortality rates. Alarmingly, the resistance rate to ciprofloxacin, used for uncomplicated urinary tract infections, varied significantly, from 8.4% to 92.9% for *E. coli* and from 4.1% to 79.4% for *K. pneumoniae* across countries reporting to the Global Antimicrobial Resistance and Use Surveillance System (GLASS) (Antibiotic resistance, World Health Organization, 2023). Therefore, the primary aim of this study was to assess the prevalence of antibiotic resistance across various wards and specimens to enhance understanding and provide suitable treatment regimens for patients.

Frequent monitoring of antibiotic usage is another recommended practice within antimicrobial stewardship programs, providing insights into usage trends. Such surveillance can highlight instances of any inappropriate use, which further necessitates further audits or interventions (Antibiotic resistance, World Health Organization, 2023). Antibiotic usage is typically measured in terms of DDDs, which standardize comparisons globally. Each antibiotic is assigned an anatomical therapeutic chemical (ATC) code and a DDD value in grams (Antibiotic resistance,World Health Organization, 2016). The WHO advised expressing DDD per 100 bed-days in hospitals and per 1,000 patient-days (PD) to accurately reflect the exact usage (WHO, 2016). The DDD can be compared among hospitals or even across countries, with those exceeding the upper limit (DDD + SD) considered high users, potentially requiring additional audits (Antibiotic resistance, World Health Organization, 2023).

Numerous studies have indicated that antibiotic usage significantly influences the prevalence of antibiotic resistance (Shapiro et al., 2020). For instance, Abdul Aziz et al. (2023) reported a 17% reduction in the mean DDD per 1,000 patient-days for antibiotics targeted by antimicrobial stewardship (AMS) programs, decreasing from 161.52 to 134.49 DDD. This reduction was primarily attributed to the decreased use of third-generation cephalosporins, carbapenems, and colistin. These findings strongly suggest that antibiotic usage directly impacts the prevalence of antibiotic resistance.

The main objective of this study was to determine the correlation between antibiotic usage across various pharmacological classes and antibiotic resistance prevalence and patterns (antibiogram) in hospitals in Penang.

## Methods

### Study design and population

This was a retrospective cross-sectional study reviewing the positive bacterial culture results from various sources and the total antibiotic usage in terms of grams or units in hospitals in Penang for 3 years from January 2021 to December 2023. A convenient sampling method was used in this study, which means that only positive bacterial culture results were included. The required data for all the positive isolates (identified microorganisms) and antibiotic usage from January 2021 to December 2023 were obtained accordingly for research purposes. The study end-point was to determine the minimum inhibitory concentration (MIC) for each pathogen against the corresponding antibiotics and classify accordingly as susceptible, intermediate, or resistant.

### Study settings

This study was conducted in six general hospitals in the state of Penang, Malaysia. These hospitals have the capacity to serve a population of 1.77 million, as revealed by the Department of Statistics Malaysia. This facility provides almost all the sub-specialty services for the entire northern region of Peninsular Malaysia.

### Inclusion and exclusion criteria

All the first positive bacterial isolates from 1 January 2021 to 31 December 2023 were included. The samples were collected from inpatients only from all wards with more than 48 hours of admission. Only one sample per patient (applicable if the same patient had >1 positive sample) was counted in this study. Varieties of biological samples were included in this study such as blood culture, cerebrospinal fluid (CSF) culture, and other body fluid cultures such as from drains and bile, pus swab culture, high vaginal swab (HVS) culture, sputum (SPT) culture, urine culture, bone and joint aspirate culture, pus fluid culture, tissue culture, and stool culture. However, those with incomplete bacterial resistance data (e.g. unidentifiable microorganism due to gross contamination) were excluded from this study.

The calculation of total antibiotic usage in terms of DDD from 1 January 2021 to 31 December 2023 was included. The DDDs were collected from inpatients only from all wards and for intravenous antibiotics only.

### Data collection

The data were extracted from the integrated computerized system. The sample size was determined using the simple formula by Daniel (1999) with a 95% confidence interval, Z value of 1.96, expected prevalence of carbapenem-resistant Enterobacterales (CREs) and extended-spectrum beta-lactamase (ESBL) at Hospital Pulau Pinang (Chuah H. C., 2019) of 0.2%, and precision of 5%. Hence, the minimum sample size required was 285 according to the formula adapted from Daniel (1999). The methods of bacterial identification adopted were Gram staining and culture-based identification, whereby the bacteria were grown on suitable media. The colony morphology, colour changes, and haemolysis patterns (on blood agar) help in preliminary identification. The antimicrobial susceptibility testing methods used were the MIC and disk diffusion method as recommended by the EUCAST 2022. Molecular method using polymerase chain reaction (PCR) was adopted to detect genes associated with antibiotic resistance, resulting in ESBL and CREs. XDR was defined as a microorganism resistant to at least one antibiotic in all but two or fewer antibiotic classes, including beta-lactams, aminoglycosides, and fluoroquinolones. The microorganism was classified as MDR if it was resistant to at least one agent in three or more antibiotic classes. CREs were defined as microorganisms resistant to carbapenem antibiotics, which were further confirmed via PCR to detect the genes responsible for the mutations (mostly NDM or OXA-48). ESBL was measured via disk diffusion or MIC testing method for antimicrobial susceptibility testing using third-generation cephalosporins + clavulanic acid.

However, the sample size for the antibiotic usage was determined using the simple formula of finite population correction considering a large population size of 1.77 million with 95% confidence interval, Z value of 1.96, standard deviation of DDD/1,000 PD (Lee et al., 2018) of ± 55.5, and margin of error of 5%. Hence, the sample size required was 473 prescriptions (Daniel, 1999). The usage of antibiotics was recorded by the respective pharmacists stationed at multiple pharmacy satellites based on the supply of antibiotics as per the inpatient prescription.

Ethical board approval from the Medical Research Ethics Committee (MREC) and the Ministry of Health (MOH) was obtained prior to the initiation of this study. The approval number given was NMRR ID-23-00308-SB6.

### Statistical analysis

Statistical evaluation was performed using IBM SPSS version 22 (SPSS Inc., Chicago, IL, USA), with a 95% confidence interval (CI) calculated accordingly, and a p-value of less than 0.05 was considered significant. For the antibiotic resistance-related analysis, descriptive analysis was performed in this study using Pearson’s chi-square (χ^2^) test. However, the prevalence was calculated using the Comprev^®^ software version 3.0.31. The formula for prevalence calculation was the number of existing cases divided by the total population with similar cases and then multiplied by 100 to yield the percentage.

For the antibiotic usage analysis, descriptive analysis was performed in this study using Friedman’s test, considering that the data were not normally distributed, and a non-parametric equivalent of repeated-measures ANOVA. The DDD was calculated by total antibiotic usage in grams divided by the DDD given by the WHO. It was further divided into the number of patient-days for that particular year (obtained from the respective admission office) and then multiplied by 1,000.


$$\text{DDD}=\frac{\text{ Total amount of drug used }(\text{in grams or another appropriate unit})}{\text{DDDs assigned by the WHO for that drug}}$$

DDD per 1000 PD=(Total DDDs of a drug used/Total patient‐days)×1000

The correlation or association between antibiotic usage and the prevalence of ESBL and CREs was determined using Pearson’s and Spearman’s correlation tests. A linear regression test was also performed to further explain the correlation.

## Results

### The prevalence and antibiotic susceptibility patterns (antibiogram)

This study was aimed primarily to determine the correlation between antibiotic usage and antibiotic resistance pattern, commonly known as antibiogram, within 3 years. The proposed hypothesis was that there is a significant association between the two variables, which means that increased DDD of the respective antibiotic class would consequently result in increased resistance capability of the associated bacterial pathogens. Therefore, this study merged the data obtained from the microbiology and pharmacy units for the antibiogram and DDD data, respectively, to further evaluate the correlation.

**Table 1** summarizes the data for each sample type across the 3 years, showing both the number of samples and their respective percentages. All p-values were below 0.05, meaning that every sample type experienced a significant shift over the years. The changes in distribution were statistically significant for each culture type.

**Table 1:** The total number and types of culture samples accepted for culture and sensitivity (C&S) in Penang for the years 2021, 2022, and 2023.

Sample type	2021 (Total) (%) n = 79,122	2022 (Total) (%) n = 84,649	2023 (Total) (%) n = 69,891	*P*-value <0.0001
Blood culture	34,896 (44.1)	42,177 (49.8)	32,473 (46.5)	<0.0001
Cerebrospinal fluid (CSF)	311 (0.4)	531 (0.6)	476 (0.7)	<0.0001
Other body fluids	1,649 (2.1)	4,816 (5.7)	1,655 (2.4)	<0.0001
Pus swab culture	4,601 (5.8)	3,616 (4.3)	5,051 (7.2)	<0.0001
High vaginal swab (HVS)	6,174 (7.8)	6,275 (7.4)	5,939 (8.5)	0.0078
Sputum (SPT)	2,886 (3.6)	4,870 (5.8)	5,442 (7.8)	<0.0001
Urine culture	11,023 (13.9)	12,664 (15.0)	11,107 (15.9)	<0.0001
Bone and joint culture	7,659 (9.7)	4,659 (5.5)	5,001 (7.2)	<0.0001
Pus culture	1,280 (1.6)	1,248 (1.5)	1,422 (2.0)	0.0015
Tissue culture	7,324 (9.3)	1,417 (1.7)	1,081 (1.5)	<0.0001
Stool culture	1,319 (1.7)	2,376 (2.7)	244 (0.3)	<0.0001

**Table 2** shows the total number of isolates for various types of microorganisms (from all samples) for 3 years in Penang. Most microorganisms mentioned above significantly changed in distribution over time. *S. pneumoniae*, CRE *E. coli*, and CRE *K. pneumoniae* did not show a significant change. Otherwise, resistance trends like methicillin-resistant *S. aureus* (MRSA), ESBL, and MDR strains showed major shifts.

**Table 2:** The total number of isolates for various types of microorganisms (from all samples) obtained in Penang for the years 2021, 2022, and 2023.

Microorganism	Total isolates (2021) (%) n = 675	Total isolates (2022) (%) n = 861	Total isolates (2023) (%) n = 1,501	*P*-value <0.0001
*Staphylococcus aureus*	163 (24.1)	178 (20.7)	380 (25.3)	<0.0001
Methicillin-resistant *S. aureus* (MRSA)	27 (4.0)	34 (3.9)	89 (5.9)	<0.0001
*Streptococcus pneumoniae*	2 (0.3)	3 (0.3)	4 (0.3)	0.7165
*Enterococcus faecalis*	12 (1.8)	19 (2.2)	40 (2.7)	<0.0001
*Enterococcus faecium*	10 (1.5)	23 (2.7)	29 (1.9)	0.0104
*Acinetobacter baumannii*	23 (3.4)	44 (5.1)	61 (4.1)	0.0002
Extensively drug-resistant (XDR) *A. baumannii*	16 (2.4)	13 (1.5)	34 (2.3)	0.0021
*Escherichia coli*	95 (14.1)	136 (15.8)	92 (6.1)	0.0037
Extended-spectrum beta-lactamase (ESBL) *E. coli*	45 (6.7)	35 (4.1)	129 (8.6)	<0.0001
Carbapenem-resistant Enterobacterales (CRE) *E. coli*	5 (0.7)	2 (0.2)	2 (0.1)	0.3679
*Klebsiella pneumoniae*	124 (18.4)	200 (23.2)	311 (20.7)	<0.0001
ESBL *K. pneumoniae*	62 (9.2)	62 (7.2)	116 (7.7)	<0.0001
CRE *K. pneumoniae*	18 (2.7)	13 (1.5)	25 (1.7)	0.1428
*Pseudomonas aeruginosa*	66 (9.7)	92 (10.7)	172 (11.5)	<0.0001
Multidrug-resistant (MDR) *P. aeruginosa*	7 (1.0)	7 (0.9)	17 (1.1)	0.0397

**Table 3** shows the number of bacterial isolates from blood cultures only for each bacterial species across the years 2021, 2022, and 2023. Three microorganisms, namely, MRSA, *K. pneumoniae*, and *P. aeruginosa*, showed significant changes in the number of total isolates in the 3 years.

**Table 3:** The total number of isolates for various types of microorganisms (from blood culture only) obtained in Penang for the years 2021, 2022, and 2023.

Microorganism	Total isolates (2021) (%) n = 395	Total isolates (2022) (%) n = 478	Total isolates (2023) (%) n = 505	*P*-value <0.0001
*Staphylococcus aureus*	104 (26.3)	94 (19.7)	98 (19.4)	0.774
Methicillin-resistant (MRSA) *S. aureus*	14 (3.5)	10 (2.1)	25 (5.0)	0.025
*Streptococcus pneumoniae*	2 (0.5)	2 (0.4)	3 (0.6)	0.867
*Enterococcus faecalis*	7 (1.8)	13 (2.7)	11 (2.2)	0.405
*Enterococcus faecium*	8 (2.0)	20 (4.2)	16 (3.2)	0.078
*Acinetobacter baumannii*	18 (4.6)	21 (4.4)	15 (3.0)	0.607
Extensively drug-resistant (XDR) *A. baumannii*	8 (2.0)	7 (1.5)	13 (2.6)	0.331
*Escherichia coli*	71 (18.0)	92 (19.3)	85 (16.8)	0.251
Extended-spectrum beta-lactamase (ESBL) *E. coli*	28 (7.1)	23 (4.8)	41 (8.1)	0.006
Carbapenem-resistant Enterobacterales (CRE) *E. coli*	4 (1.0)	2 (0.4)	2 (0.4)	0.607
*Klebsiella pneumoniae*	74 (18.7)	115 (24.1)	91 (18.0)	0.011
ESBL *K. pneumoniae*	35 (8.9)	27 (5.7)	32 (6.3)	0.594
CRE *K. pneumoniae*	14 (3.5)	9 (1.9)	20 (4.0)	0.120
*Pseudomonas aeruginosa*	6 (1.5)	40 (8.4)	50 (9.9)	<0.0001
Multidrug-resistant (MDR) *P. aeruginosa*	2 (0.5)	3 (0.6)	3 (0.6)	0.882

The most common types of Gram-positive microorganisms isolated were *S. aureus*, MRSA, *S. pneumoniae*, *Enterococcus faecalis*, and *Enterococcus faecium*. **Table 4** depicts the prevalence of various microorganisms from all samples obtained in Penang for 3 years. Statistically significant differences were observed for most microorganisms, except for *S. pneumoniae*, carbapenem-resistant Enterobacterales *E. coli*, and carbapenem-resistant Enterobacterales *K. pneumoniae* across the study span of 3 years. **Table 5** shows the prevalence of multiple microorganisms from blood culture only in the 3 years. *P. aeruginosa* is the most concerning, showing a statistically significant rise over time.

**Table 4:** The prevalence of various types of microorganisms (from all samples) obtained in Penang for the years 2021, 2022, and 2023.

Microorganism	Total isolates (2021) (Prevalence) n = 79,122	Total isolates (2022) (Prevalence) n = 84,649	Total isolates (2023) (Prevalence) n = 69,891	*P*-value <0.0001
*Staphylococcus aureus*	163 (0.2)	178 (0.2)	380 (0.5)	<0.0001
Methicillin-resistant (MRSA) *S. aureus*	27/163 (16.6)	34/178 (19.1)	89/380 (23.4)	<0.0001
*Streptococcus pneumoniae*	2 (0.003)	3 (0.004)	4 (0.006)	0.717
*Enterococcus faecalis*	12 (0.02)	19 (0.02)	40 (0.06)	<0.0001
*Enterococcus faecium*	10 (0.01)	23 (0.03)	29 (0.04)	0.0104
*Acinetobacter baumannii*	23 (0.030	44 (0.05)	61 (0.09)	0.0002
Extensively drug-resistant (XDR) *A. baumannii*	16/23 (69.6)	13/44 (29.5)	34/61 (55.7)	0.0021
*Escherichia coli*	95 (0.12)	136 (0.16)	290 (0.41)	<0.0001
Extended-spectrum beta-lactamase (ESBL) *E. coli*	45/95 (47.4)	35/136 (25.7)	129/290 (44.5)	<0.0001
Carbapenem-resistant Enterobacterales (CRE) *E. coli*	5/95 (5.26)	2/136 (1.47)	5/290 (1.72)	0.472
*Klebsiella pneumoniae*	124 (0.16)	200 (0.24)	311 (0.44)	<0.0001
ESBL *K. pneumoniae*	62/124 (50.0)	62/200 (31.0)	116/311 (37.3)	<0.0001
CRE *K. pneumoniae*	18/124 (14.5)	13/200 (6.5)	25/311 (8.0)	0.143
*Pseudomonas aeruginosa*	66 (0.08)	92 (0.11)	172 (0.25)	<0.0001
Multidrug-resistant (MDR) *P. aeruginosa*	7/66 (10.6)	7/92 (7.6)	17/172 (9.9)	0.0397

**Table 5:** The prevalence of various types of microorganisms (from blood culture only) obtained in Penang for the years 2021, 2022, and 2023.

Microorganism	Total isolates (2021) (Prevalence) n = 34,896	Total isolates (2022) (Prevalence) n = 42,177	Total isolates (2023) (Prevalence) n = 32,473	*P*-value <0.0003
*Staphylococcus aureus*	104 (0.3)	94 (0.2)	98 (0.3)	0.048
Methicillin-resistant (MRSA) *S. aureus*	14/104 (13.5)	10/94 (10.6)	25/98 (25.5)	0.059
*Streptococcus pneumoniae*	2 (0.006)	2 (0.005)	3 (0.009)	0.677
*Enterococcus faecalis*	7 (0.02)	13 (0.03)	11 (0.03)	0.384
*Enterococcus faecium*	8 (0.02)	20 (0.05)	16 (0.05)	0.205
*Acinetobacter baumannii*	18 (0.05)	21 (0.05)	15 (0.05)	0.419
Extensively drug-resistant (XDR) *A. baumannii*	8/18 (44.4)	7/21 (33.3)	13/15 (86.7)	0.245
*Escherichia coli*	71 (0.20)	92 (0.22)	85 (0.26)	0.229
Extended-spectrum beta-lactamase (ESBL) *E. coli*	28/71 (39.4)	23/92 (25.0)	41/85 (48.2)	0.125
Carbapenem-resistant Enterobacterales (CRE) *E. coli*	4/71 (5.63)	2/92 (2.17)	2/85 (2.35)	0.542
*Klebsiella pneumoniae*	74 (0.21)	115 (0.27)	91 (0.28)	0.078
ESBL *K. pneumoniae*	35/74 (47.3)	27/115 (23.5)	32/91 (35.2)	0.170
CRE *K. pneumoniae*	14/74 (18.9)	9/115 (7.8)	20/91 (22.0)	0.155
*Pseudomonas aeruginosa*	6 (0.02)	40 (0.09)	50 (0.15)	<0.001
Multidrug-resistant (MDR) *P. aeruginosa*	2/6 (33.3)	3/40 (7.5)	3/50 (6.0)	0.345

**Tables 6** and **7** illustrate further the various types of Gram-positive and Gram-negative microorganisms, respectively, across the years. Almost all the microorganisms showed no difference in terms of significant antibiotic susceptibility, except for *K. pneumoniae*, which had significant changes in susceptibility over the years, especially for cefoperazone/sulbactam, amoxicillin/clavulanic acid, ceftriaxone, and ceftazidime.

**Table 6:** Antibiogram table for Gram-positive microorganisms obtained in Penang from year 2021 to year 2023.

Antibiotic tested	Susceptibility (%)			*P*-value
	Year 2021	Year 2022	Year 2023	
*Staphylococcus aureus*				
Cefazolin	100	95	95	0.9174
Cloxacillin	100	95	95	0.9174
Rifampicin	95	90	100	0.7686
Sulfamethoxazole/trimethoprim	95	94	95	0.9965
Clindamycin	85	91	90	0.8899
Fusidic acid	80	73	80	0.8103
Methicillin-resistant *S. aureus* (MRSA)				
Vancomycin	82	85	81	1.00
Rifampicin	100	90	89	1.00
Sulfamethoxazole/trimethoprim	100	89	85	1.00
Clindamycin	95	75	82	1.00
Fusidic acid	85	71	74	1.00
*Streptococcus pneumoniae*				
Oxacillin	100	55	100	1.00
Ceftriaxone	100	100	100	1.00
Sulfamethoxazole/trimethoprim	50	100	75	1.00
Clindamycin	100	100	100	1.00
Erythromycin	100	67	70	1.00
*Enterococcus faecalis*				
Ampicillin	90	91	84	0.787
*Enterococcus faecium*				
Vancomycin	90	95	100	0.979

**Table 7:** Antibiogram table for Gram-negative microorganisms obtained in Penang from year 2021 to year 2023.

Antibiotic tested	Susceptibility (%)			*P*-value
	Year 2021	Year 2022	Year 2023	
*Acinetobacter baumannii*				
Ampicillin/sulbactam	92	73	72	1.00
Cefoperazone/sulbactam	85	69	85	1.00
*Escherichia coli*				
Ampicillin/sulbactam	65	53	59	0.681
Cefoperazone/sulbactam	95	91	99	0.917
Amoxicillin/clavulanic acid	62	55	59	0.875
Ceftriaxone	95	94	94	0.998
Cefoperazone	89	88	84	0.958
Cefuroxime	82	83	82	0.998
Ceftazidime	91	93	92	0.994
Sulfamethoxazole/trimethoprim	72	58	63	0.624
*Klebsiella pneumoniae*				
Ampicillin/sulbactam	77	77	83	0.1685
Cefoperazone/sulbactam	99	93	99	0.0001
Amoxicillin/clavulanic acid	75	73	87	0.0001
Ceftriaxone	100	95	99	0.0016
Cefoperazone	95	92	91	0.3768
Cefuroxime	89	85	91	0.1113
Ceftazidime	98	91	99	<0.0001
Sulfamethoxazole/trimethoprim	88	82	89	0.0672
*Pseudomonas aeruginosa*				
Cefoperazone	90	87	85	0.9621
Cefepime	94	88	83	0.8336
Ceftazidime	95	93	92	0.9872
Piperacillin/tazobactam	98	82	74	0.3908
Ciprofloxacin	97	96	91	0.9450

### Total defined daily dose for the total antibiotics used

This study presents the trends in the use of various antibiotics, as measured by DDDs per 1,000 PD, over 3 years (2021–2023). The data reveal notable variations in the usage of certain antibiotic classes and specific drugs, which could reflect changes in clinical practice, resistance patterns, or hospital policies.

A total of 689 prescriptions within the study duration that met the inclusion and exclusion criteria were included in this study. The results in **Table 8** showed a consistent increase in the usage of penicillins and cephalosporins, while other antibiotic classes such as carbapenems and fluoroquinolones demonstrated variable trends. The most obvious increment was observed in amoxicillin/clavulanic acid and ampicillin/sulbactam, both of which exhibited a steady rise in DDD per 1,000 PD from 2021 to 2023. Additionally, cephalosporins, particularly cefuroxime, ceftazidime, and ceftriaxone, showed a notable upward trend, with ceftriaxone increasing from 108.68 DDD/1,000 PD in 2021 to 131.50 DDD/1,000 PD in 2023.

**Table 8:** Total defined daily dose (DDD)/1,000 patient-days (PD) for antibiotics used in hospitals in Penang for the years 2021, 2022, and 2023 (n = 689).

Antibiotic		DDD	2021	2022	2023	*P*-value
		As per WHO	Total DDD/1,000 PD	Total DDD/1,000 PD	Total DDD/1,000 PD	
Penicillins	Amoxicillin/clavulanic acid	1 g per day (amoxicillin component)	325.60	475.50	520.70	0.368
	Ampicillin/sulbactam	1 g per day (ampicillin component)	780.00	855.10	1,069.00	0.368
	Piperacillin/tazobactam	3 g per day (piperacillin component)	170.30	268.30	287.60	0.368
Cephalosporins	Cefazolin	3 g per day	44.30	64.50	55.38	0.368
	Cefuroxime	1.5 g per day	125.70	180.20	217.60	0.368
	Ceftazidime	6 g per day	63.04	80.25	105.76	0.368
	Cefoperazone	4 g per day	22.98	28.06	26.79	0.368
	Cefoperazone/sulbactam	4 g per day (cefoperazone component)	0.21	0.82	0.77	0.368
	Ceftriaxone	2 g per day	108.68	126.78	131.50	0.368
	Cefotaxime	2 g per day	0.95	1.26	1.80	0.368
	Cefepime	6 g per day	42.83	44.71	63.53	0.368
	Ceftaroline	2 g per day	0.13	0.25	0.43	0.368
Carbapenem	Ertapenem	1 g per day	1.04	0.45	1.53	0.368
	Meropenem	3 g per day	51.92	57.79	89.97	0.368
	Imipenem	1 g per day (imipenem component)	4.30	4.89	4.19	0.368
Glycopeptides	Vancomycin	2 g per day	15.88	18.41	20.28	0.368
Oxazolidinone	Linezolid	600 mg per day	0.18	1.02	0.43	0.368
Glycylcycline	Tigecycline	100 mg per day	0.00	0.00	0.11	0.368
Polymyxin	Polymyxin E	150 mg per day (based on colistin)	7.11	6.61	8.84	0.368
	Polymyxin B	25 mg per day	0.04	1.01	0.20	0.368
Fluoroquinolone	Ciprofloxacin	1 g per day	7.11	6.47	12.22	0.368
Total DDD			1,772.30	2,222.38	2,618.63	<0.0001

Chi-square statistic (χ^2^) = 20.78; degrees of freedom (df) = 2; p-value = 0.0041.

However, carbapenems showed mixed results. Meropenem usage increased substantially from 51.92 DDD/1,000 PD in 2021 to 89.97 DDD/1,000 PD in 2023. Conversely, ertapenem showed a decline in usage, with only slight fluctuations over the 3 years, a statistically significant difference in total antibiotic usage across the 3 years, which suggests that at least 1 year, it had a significantly different antibiotic consumption compared to the others [chi-square statistic (χ^2^) = 20.78; degrees of freedom (df) = 2; p-value = 0.0041]. However, there was no statistically significant difference in the usage of each type of antibiotic across the years 2021, 2022, and 2023.

### Correlation between antibiotic usage and antibiotic resistance pattern

In terms of the correlation between total third-generation cephalosporin usage, including ceftazidime, cefoperazone, cefoperazone/sulbactam, ceftriaxone, and cefotaxime, and ESBL, Pearson’s correlation was 0.777, which suggests a strong positive correlation, but the p-value (0.433) indicated that the correlation was not statistically significant. Spearman’s correlation was 0.500, which suggests a moderate positive correlation, but again, the p-value (0.667) indicated a lack of statistical significance. However, the linear regression R^2^ value shows 0.604, which means that approximately 60.4% of the variation in ESBL cases can be explained by third-generation cephalosporin usage, although the p-value (0.433) was still not significant. **Table 9** shows the correlation results across 3 years in Penang.

**Table 9:** Correlation between third-generation cephalosporin usage and prevalence of extended-spectrum beta-lactamase (ESBL)-producing *Escherichia coli* and *Klebsiella pneumoniae* in hospitals in Penang for the years 2021, 2022, and 2023.

2021		2022		2023		Correlation coefficient	*P*-value
DDD	ESBL	DDD	ESBL	DDD	ESBL		
195.86	107	237.17	97	266.62	245	Pearson’s coefficient: 0.777	0.433
Spearman’s correlation: 0.500	0.667						

Defined daily dose (DDD) includes ceftazidime, cefoperazone, cefoperazone/sulbactam, ceftriaxone, and cefotaxime only.

**Table 10** shows the correlation results between carbapenem usage and the prevalence of CREs across 3 years in Penang. Pearson’s correlation was 0.762, which suggests a strong positive correlation, but the p-value (0.448) indicated that the correlation was not statistically significant. Spearman’s correlation was 0.500, which suggests a moderate positive correlation, but again, the p-value (0.667) indicated a lack of statistical significance. However, the linear regression R^2^ value shows 0.581, which means that approximately 58.1% of the variation in CRE cases can be explained by carbapenem usage, although the p-value (0.537) was still not significant.

**Table 10:** Correlation between carbapenem usage and prevalence of carbapenem-resistant *Enterobacterales* (CRE) *Escherichia coli* and *Klebsiella pneumoniae* in hospitals in Penang for the years 2021, 2022, and 2023.

2021		2022		2023		Correlation coefficient	*P*-value
DDD	CRE	DDD	CRE	DDD	CRE		
57.26	23	63.13	15	9,569	30	Pearson’s coefficient: 0.762	0.448
						Spearman’s correlation: 0.500	0.667

Defined daily dose (DDD) includes meropenem, imipenem, and ertapenem only.

## Discussion

This study investigated the prevalence of resistant bacterial isolates from all samples and blood cultures over a period of 3 years (2021–2023) in several hospitals. The results reveal significant findings regarding both the prevalence of bacterial isolates and antibiotic resistance patterns. These findings contribute to understanding the current state of all samples’ culture susceptibility in both Gram-positive and Gram-negative microorganisms in Penang.

Globally, the positivity rate of blood cultures in this study (12%–13%) was within the accepted range, aligning with findings from global studies, such as those by Kahlmeter et al. (2020), which reported positivity rates of 10%–14% in similar hospital settings. However, the issue of insufficient blood volume affecting positivity rates has been identified as a critical factor in improving diagnostic yield, as shown in quality assurance initiatives in the UK (Bookstaver et al., 2017). The steps being taken in the current study via a quality assurance project to improve blood collection methods could lead to better outcomes, as studies by Zhang et al. (2023) have suggested that increasing blood sample volumes significantly improves positivity rates.

The study found that the number of bacterial isolates has steadily increased, with the prevalence of microorganisms rising from all samples and blood culture only in 2021 to 2023, with p-values of p < 0.0001 and p < 0.0003, respectively. This upward trend is consistent with findings from other studies. For example, a study by Ahmed et al. (2022) in South Asia reported a similar rise in bacterial isolate prevalence over 5 years, which they attributed to the increasing use of antibiotics and the changing microbial landscape in hospitals. The increase in isolates in the present study can be linked to multiple factors, including improved diagnostic technologies and, perhaps, concerningly, the injudicious use of antibiotics, which could contribute to higher bacterial resistance rates (Vijayakumar et al., 2020). However, there is a possibility that the total number of bacterial isolates during the COVID-19 pandemic, compared to the previous years (e.g. 2018), showed significant fluctuations, which could be due to changes in healthcare practices, infection control measures, and antibiotic usage. Several studies have reported a decline in overall bacterial isolates from clinical samples, largely attributed to reduced hospital visits, widespread use of masks, social distancing, and improved hygiene practices, which limited the transmission of bacterial infections. A study in Finland observed a decrease in the prevalence of *E. coli* isolates in urinary tract and bloodstream infections during the pandemic (Ilmavitra et al., 2024).

Notably, the increasing prevalence of MRSA in this study is consistent with reports from other regions, such as the USA and Europe, where MRSA rates often range from 20% to 30% (Klein et al., 2020). The fact that all MRSA isolates were >80% sensitive to vancomycin is a positive sign, in contrast to studies indicating increasing vancomycin resistance in some regions, such as India, where vancomycin-resistant *S. aureus* (VRSA) has been reported at rates higher than 5% (Wu et al., 2021). Moreover, the gradual development of resistance to rifampicin and trimethoprim/sulfamethoxazole in MRSA isolates (10%–20%) noted in this study mirrors the findings of a similar study in the South Korea by Bae et al., (2023), who found a rising trend in rifampicin resistance in MRSA isolates, which poses a challenge for treatment, especially for oral treatments.

In terms of *E. faecalis*, this study found that its isolates are gradually becoming resistant to ampicillin, which is concerning. Similar trends have been observed globally, as shown by studies such as those by Daniel et al. (2015), who reported high resistance rates to ciprofloxacin among enterococci in Southeast Asia. The gradual resistance to ampicillin in *E. faecalis* in this study is also consistent with global patterns, where studies in the USA and Europe have noted a steady increase in ampicillin resistance over the past decade (Zhang et al., 2021).

The study’s findings on Gram-negative bacteria, particularly *E. coli*, *A. baumannii*, and *P. aeruginosa*, reflect a concerning rise in resistance rates. *E. coli* demonstrated resistance to ampicillin/sulbactam and amoxicillin/clavulanic acid, with an alarming rise in CREs. This aligns with global reports, such as those from the World Health Organization (2021), which have highlighted the increasing spread of CREs worldwide, particularly in healthcare settings. The high prevalence of carbapenem resistance in *E. coli* and *K. pneumoniae* strains found to be a growing concern due to the limited treatment options for such infections. A similar study in India noted that the prevalence of CREs has increased significantly over the past few years, echoing the findings in this study (Devi et al., 2024).

The finding that *A. baumannii* is acquiring resistance to ampicillin/sulbactam is also supported by international reports. Studies conducted in South Korea have reported that *Acinetobacter baumanii* strains are increasingly resistant to common beta-lactams, with XDR strains becoming more prevalent (Kang et al., 2024). The fact that *A. baumannii* remains sensitive to polymyxin B in this study is promising, but global surveillance has warned of the potential for polymyxin resistance emerging in the future, which could complicate treatment (Cai et al., 2012).

Similarly, *P. aeruginosa* remains sensitive to ceftazidime and ciprofloxacin, which is a positive finding, despite gradually developing resistance to cefepime and piperacillin/tazobactam (preferred options in this facility), as resistance in *P. aeruginosa* has been steadily rising worldwide, as per the recent report from Europe (Oliver et al., 2015).

In this study, the total DDD/1,000 PD for the antibiotics used revealed a significant difference across 3 years, which is parallel with numerous studies conducted worldwide (Zhong et al., 2021; Chuah et al., 2019). The increase in the DDD of amoxicillin/clavulanic acid and ampicillin/sulbactam may reflect an escalation in infections requiring broad-spectrum beta-lactamase inhibitors, possibly due to the increasing prevalence of beta-lactamase-producing organisms (Bookstaver et al., 2017). These findings align with global concerns about antibiotic resistance, where beta-lactamase inhibitors are often prescribed to combat resistant strains, including those producing ESBLs (Chambers and DeLeo, 2020).

However, the consistent rise in the use of cephalosporins, particularly cefuroxime, ceftazidime, and ceftriaxone, may indicate an increased clinical need for agents targeting a broad range of bacterial pathogens, including those associated with respiratory, intra-abdominal, and urinary tract infections (Wushouer et al., 2023). These findings suggest that hospitals may be adopting broader-spectrum cephalosporins as empirical therapy, likely due to changes in prescribing practices.

In contrast, the usage trends for carbapenems (notably meropenem) may signal an increased reliance on these agents as last-line therapies for ESBL organisms. The notable increase in meropenem use, specifically, could be due to rising cases of multidrug-resistant Gram-negative microorganisms like ESBL-producing *E. coli* and *K. pneumoniae* (Livermore, 2021). While ertapenem remained relatively stable with a slight decline, the increase in meropenem usage suggests a shift towards using carbapenems in more severe or resistant infections, possibly due to the inability of other antibiotics to treat these resistant strains (e.g. *P. aeruginosa*).

In a nutshell, the findings in this study are consistent with previous studies showing an increase in the use of broad-spectrum antibiotics, particularly in the context of antibiotic resistance. For example, an increase in the use of cephalosporins and penicillins with beta-lactamase inhibitors has been observed in other hospital settings, where empirical treatments often favour these agents to cover a wider range of pathogens (Paterson et al., 2022). The rise in the use of meropenem mirrors trends seen in global healthcare settings, where the growing prevalence of carbapenem-resistant organisms has led to an increased need for these last-line agents (Zhong et al., 2021).

The trends observed in this study regarding the correlation between antibiotic usage and antibiotic resistance rates align with those seen in other healthcare settings globally, where the antibiotic resistance rate has risen because of increased usage. It is parallel with findings in another recent study, which has also shown a growing reliance on broad-spectrum antibiotics, particularly beta-lactam and carbapenem-based therapies, due to the increasing prevalence of multidrug-resistant pathogens (Paterson et al., 2022). Studies have noted that ESBL-producing microorganisms are driving up the use of carbapenems and other broad-spectrum antibiotics in many healthcare settings (Livermore, 2021). The increase in the use of cephalosporins and penicillins with beta-lactamase inhibitors observed here is consistent with global trends indicating the escalating treatment costs associated with resistant infections (Klein et al., 2020).

The rise in antibiotic resistance is driven by multiple mechanisms, including the overuse and misuse of antibiotics in both healthcare and agriculture, leading to selective pressure for resistant strains (Livermore, 2021). Horizontal gene transfer (HGT), involving plasmids and transposons, facilitates the spread of resistance genes such as ESBL and carbapenemases among bacterial populations (Zhang et al., 2021). Additionally, bacterial mutations and biofilm formation contribute to increased resistance, particularly in hospital-acquired infections (Ahmed et al., 2022). The COVID-19 pandemic further exacerbated antibiotic resistance due to the widespread empirical use of antibiotics for suspected secondary bacterial infections, despite bacterial co-infection rates being less than 10% (Devi et al., 2022). Hospitals experienced disruptions in antimicrobial stewardship programs, leading to increased prescriptions of broad-spectrum antibiotics like carbapenems and fluoroquinolones, which likely contributed to rising rates of multidrug-resistant organisms (World Health Organization, 2021). Furthermore, prolonged hospital stays, increased ventilator use, and inadequate infection control during the pandemic created an environment conducive to the spread of resistant bacteria, including MRSA and CREs (Oliver et al., 2015). These findings highlight the urgent need for stronger antimicrobial stewardship programs and global surveillance to mitigate the escalating antibiotic resistance crisis.

## Conclusion

The correlation between the use of third-generation cephalosporins and ESBL rate, as well as the use of carbapenems and CRE rate, further suggests that controlling certain antibiotic usage could help mitigate the rising AMR. The key findings of this study revealed a concerning gradual rise in resistant bacterial isolates in both Gram-positive and Gram-negative microorganisms. The increase in resistance, especially against commonly used antibiotics like penicillins and cephalosporins, highlights the growing challenge of managing infections, especially multidrug-resistant pathogens like ESBL and CREs. The study’s findings align with global trends, reinforcing the need for more effective strategies in combating AMR, particularly in healthcare settings.

## Strengths and limitations

The study centres are among the main referral centres, especially in the northern region of Peninsular Malaysia, covering almost all the major sub-specialties and thus being considered the major strength of this study. On top of that, another added advantage is that there are three in-house infectious disease consultants, comparable to other tertiary care centres worldwide. Another strength of the study is the large number of samples pooled from the six general hospitals.

While the data presented here offer valuable insights, there are several limitations that should be considered. The study is limited by the lack of detailed patient demographic information, including the diagnosis and antibiotic history, which could further inform the contextual factors influencing the antibiotic resistance patterns.

Not only that, but the study is also limited to the analysis of DDD/1,000 PD, which may not capture the full spectrum of antibiotic prescribing practices, such as treatment duration, dose adjustments, or patient outcomes. Additionally, the data did not account for factors like the clinical severity of infections or the emergence of AMR, which could influence prescribing behaviour.

## Data Availability

The raw data supporting the conclusions of this article will be made available by the authors, without undue reservation.
