# Correction: The correlation between antibiotic usage and antibiotic resistance: a 3-year retrospective study

**DOI:** 10.3389/fcimb.2025.1678273

**Published:** 2025-08-22

**Authors:** Ann Lisa Arulappen, Amer Hayat Khan, Syed Shahzad Hasan, Sabariah Noor Harun, Ting Soo Chow, Mahmood Basil A. Al-Rawi, Wajid Syed

**Affiliations:** ^1^ Discipline of Clinical Pharmacy, School of Pharmaceutical Sciences, University Science Malaysia, Georgetown, Malaysia; ^2^ Department of Pharmacy, Penang Hospital, Ministry of Health, George, Town, Malaysia; ^3^ Department of Pharmacy, School of Applied Science, University of Huddersfield, Huddersfield, United Kingdom; ^4^ Department of Medicine, Penang Hospital, Ministry of Health, Georgetown, Malaysia; ^5^ Department of Optometry, College of Applied Medical Sciences, King Saud University, Riyadh, Saudi Arabia; ^6^ Department of Clinical Pharmacy, College of Pharmacy, King Saud University, Riyadh, Saudi Arabia

**Keywords:** antimicrobial resistance, antibiogram, gram positive, gram negative, prevalence, antibiotic usage

Author Mahmood Basil A. Al-Rawi was erroneously assigned to affiliation 1. This affiliation has now been removed for author Mahmood Basil A. Al-Rawi.

The original version of this article has been updated.

In the published article, there was an error in the **Acknowledgement** statement. “All the authors would like to express their gratitude to the Director General of Health for granting permission to publish this article and to those who have contributed directly and indirectly to completing this study. The authors of this study extend their appreciation to the Researchers Supporting Project (Project number RSP2025R378), King Saud University, Riyadh, Saudi Arabia”. The correct Acknowledgement statement appears below.

“All the authors would like to express their gratitude to the Director General of Health for granting permission to publish this article and to those who have contributed directly and indirectly to completing this study. The authors of this study extend their appreciation to the Researchers Supporting Project (ORF-2025-378), King Saud University, Riyadh, Saudi Arabia.”

The original article has been updated.

