# Correction: The correlation between antibiotic usage and antibiotic resistance: a 3-year retrospective study

**DOI:** 10.3389/fcimb.2025.1692095

**Published:** 2025-09-08

**Authors:** Ann Lisa Arulappen, Amer Hayat Khan, Syed Shahzad Hasan, Sabariah Noor Harun, Ting Soo Chow, Mahmood Basil A. Al-Rawi, Wajid Syed

**Affiliations:** ^1^ Discipline of Clinical Pharmacy, School of Pharmaceutical Sciences, University Science Malaysia, Georgetown, Malaysia; ^2^ Department of Pharmacy, Penang Hospital, Ministry of Health, George, Town, Malaysia; ^3^ Department of Pharmacy, School of Applied Science, University of Huddersfield, Huddersfield, United Kingdom; ^4^ Department of Medicine, Penang Hospital, Ministry of Health, Georgetown, Malaysia; ^5^ Department of Optometry, College of Applied Medical Sciences, King Saud University, Riyadh, Saudi Arabia; ^6^ Department of Clinical Pharmacy, College of Pharmacy, King Saud University, Riyadh, Saudi Arabia

**Keywords:** antimicrobial resistance, antibiogram, gram positive, gram negative, prevalence, antibiotic usage

An incorrect **Funding** statement was provided. The correct funder is “Ongoing Research Funding Program”. The correct funding statement reads:

“This work was funded by the Ongoing Research Funding Program (ORF-2025-378), King Saud University, Riyadh 11451, Saudi Arabia”.

The original version of this article has been updated.

There was an error in the **Acknowledgments**, the correct statement reads:

“All the authors would like to express their gratitude to the Director General of Health for granting permission to publish this article. The authors of this study extend their appreciation to the Ongoing Research Funding Program (ORF-2025-378), King Saud University, Riyadh, Saudi Arabia for their encouragement and assistance”.

The original version of this article has been updated.

